# A Hands-On Exercise on Caries Diagnostics among Dental Students—A Qualitative Study

**DOI:** 10.3390/dj9100113

**Published:** 2021-09-28

**Authors:** Heidi Kangas, Saujanya Karki, Tarja Tanner, Anne Laajala, Helvi Kyngäs, Vuokko Anttonen

**Affiliations:** 1 Research Unit of Oral Health Sciences, University of Oulu, P.O. Box 5281, 90014 Oulu, Finland; heidi.kangas@student.oulu.fi (H.K.); saujanya.karki@oulu.fi (S.K.); tarja.tanner@oulu.fi (T.T.); anne.laajala@oulu.fi (A.L.); 2 Medical Research Center, Oulu University Hospital, University of Oulu, P.O. Box 21, 90029 Oulu, Finland; helvi.kyngas@oulu.fi; 3 Research Unit of Nursing Sciences and Health Management, University of Oulu, P.O. Box 5281, 90014 Oulu, Finland

**Keywords:** dental caries, dental education, dental students, diagnosis

## Abstract

According to current care practices, the aim is to prevent the onset of caries lesions and to stop the progression of incipient lesions. A visual lesion assessment system, International Caries Detection and Assessment System (ICDAS), has been developed to promote reliability and repeatability of assessment of different stage caries lesions. The aims of this study were to evaluate the experiences of a hands-on exercise with authentic teeth as an adjunct to lecturing among third-year dental students and to evaluate the learning process during the hands-on exercise measured by qualitative (inductive content) analysis of the given feedback. In 2018, 51 third-year dental students at the University of Oulu, Finland, participated in a hands-on exercise on caries detection, where they assessed the depth and activity of lesions in extracted teeth using the ICDAS classification. After the lecture, students evaluated the exercise, giving feedback according to five given topics, three of which were analyzed using inductive content analysis. The exercise was considered useful and necessary but, overall, also challenging. The diverse activities and materials, as well as observational methods, promoted learning. The classification of lesions, the diagnostic methods, and the fact that there was not enough time to adopt things during the exercise were found to be challenging. For developing the exercise, the students suggested that more time should be scheduled for it and there should be more individual teaching. This qualitative study showed that, despite the challenge in caries diagnostics, dental students perceive the hands-on exercise as both a communal and individual learning experience.

## 1. Introduction

Dental caries is one of the most common noncommunicable disease. On the other hand, caries is unevenly distributed in the population, i.e., it is polarized, especially in the developed countries [1]. According to the Health 2011 survey, one in five Finns in northern and southern Finland needed operative care for dental caries [2]. In the Conscripts’ Oral Health Research in 2011, almost half of the conscripts were in need of operative caries treatment [3]. Caries is also common among children [4]. According to current caries care guidelines, it is important to control caries, i.e., to prevent occurrence of caries lesions and to stop the progression of incipient lesions [5]. This treatment protocol emphasizes the importance of identifying the individual risk factors of caries and detecting all lesions, including the incipient ones, and their activity as early as possible. The synthesis comprises means to influence the causes for the condition and to arrest the progression of incipient lesions or treat operatively the lesions which cannot be arrested [5].

Methods of detecting caries lesions are introduced to dental students as advised by Sculte and colleagues [6]. Lectures are the most common form of teaching in the University of Oulu, Finland, including the classifications of visual–tactile examinations, in adjunct with, e.g., fiber optic transillumination (FOTI) and radiography. Clinically, the biggest challenge for students is to detect initial caries lesions before invasive treatment is needed [7]. Visual examination usually has low sensitivity, and consequently, many lesions may go unnoticed. To improve the quality of visual examination, the ICDAS (International Caries Detection and Assessment System) classification has been developed based on which clinical signs can be used to assess the depth of lesions as well as their activity [8]. Studies have shown that the ICDAS improves distinctly the detection of caries lesions on the occlusal surfaces [9]. Similarly, the ability of dental students to assess the depth of caries lesions in extracted teeth according to ICDAS criteria after the lecture and hands-on exercise was found to be good (sensitivity 78%, specificity 87%) [7].

The aim of this study was to evaluate the experiences of a hands-on exercise with authentic teeth as an adjunct to lecturing among third-year dental students. Another aim was to evaluate the learning process during the hands-on exercise measured by qualitative (inductive content) analysis of the given feedback.

## 2. Materials and Methods

In the autumn of 2018, third-year dental students at the University of Oulu, Finland, were taught caries diagnostics through lectures followed by a hands-on exercise. Prior to the exercise, the lecture was summarized, covering the signs for estimating the depth and activity of different stages of caries lesions using the ICDAS criteria. In the hands-on exercise, there were 51 extracted premolar and molar teeth boiled in water and preserved in alcohol, each with at least one clinically visible enamel or dentinal lesion. A clinical digital photograph and an X-ray of each tooth had been taken before the exercise.

All the students examined the same 51 teeth with different stage caries lesions individually. At first, the students independently assessed the depth and activity of all the lesions in clinical photographs. After that, each student independently assessed the depth and activity of caries lesions of all 51 extracted teeth using visual–tactile examination and ICDAS criteria (0–6) and activity +/−. Students had the opportunity to use FOTI during the inspection. Simultaneously, they also evaluated the depth of the lesions on X-rays (mid-enamel/dento–enamel junction or outer/mid/deep dentin).

After everyone had evaluated all the teeth, each student split one tooth into two halves so that the caries lesion was visible. Each student introduced the clinically observed and radiographic findings of the tooth and evaluated the actual depth and activity of the lesion on the split tooth. They shared the clinical photographs and X-rays findings of each tooth to each other one by one using a data projector and also displayed the finding of the split tooth using the endo microscope (Leica M320 Dental Microscope, Leica Microsystems, Wetzlar, Germany). When one student was sharing the findings, the other students followed the presentations on large screens. The student also suggested a treatment plan (preventive or operative) for the lesion. The professor of cariology acted as a gold standard and gave her own opinion at the end of each student’s presentation. During the exercise, the split teeth were photographed again using the endo microscope, and all material was available to the students after the teaching session.

At the end of the day, students provided feedback on the hands-on exercise on the Socrative answer platform. They were asked: 1. What helped you to learn about caries diagnostics according to the ICDAS criteria (free comments), 2. What was difficult in the exercise (free comments), 3. Was the diagnostic exercise necessary in addition to the lectures (y/n, free comments), 4. Should the exercise be developed, how (y/n, free comments) 5. What did you think about the exercise (free comments). An analysis was conducted on the contents of the answers related to questions 1, 2, and 4.

Student feedback was analyzed using inductive content analysis [10], assessing saturation for each question. Open codes were generated from open responses. Open codes indicated the core content of an open answer. The contents of the open codes were compared with each other, and the codes with similar content were combined into subcategories, which were given a name descriptive of the contents. By the same principle, the subcategories were combined into main categories. From the open-ended answers to questions 1, 2, and 4, three main categories were formed. In addition to the qualitative analysis, the subcategories were quantified by calculating how many of the subjects expressed the issue mentioned in each subcategory.

## 3. Results

The response rate to the survey was excellent (96%), with 49 out of 51 students responding to the feedback form on the caries diagnostics exercise. Inductive content analysis was performed on the answers to questions 1, 2, and 4. To question 3, 44 students answered that the exercise was useful in addition to the lectures, 3 did not answer and 1 thought that the lecture and/or the exercise alone would have been sufficient. In question 5, the response was an adjective (one word). A total of 42 of the responses were considered positive and 7 negative. For questions 3 and 5, no further analysis was necessary.

**Figure 1:**
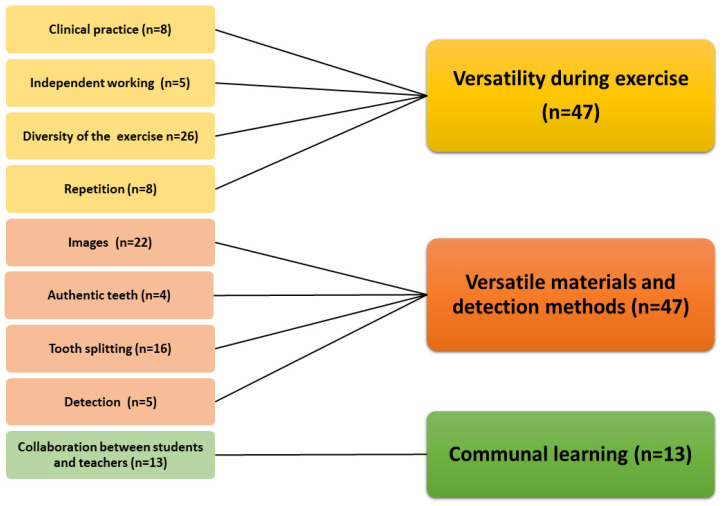
The topics that helped third-year dental students to learn ICDAS caries diagnostics criteria in the hands-on exercise.

**Figure 2:**
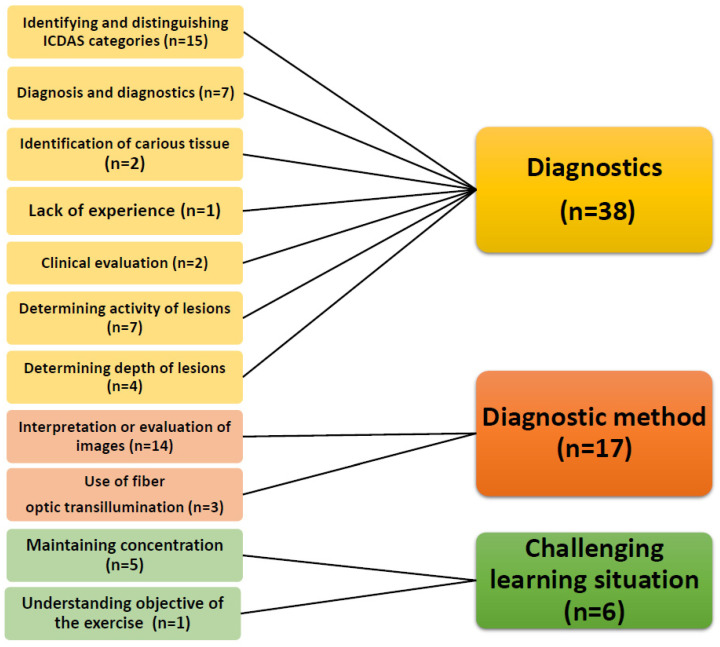
The most challenging issues in the hands-on exercise to introduce third-year dental students to caries diagnostics.

**Figure 3:**
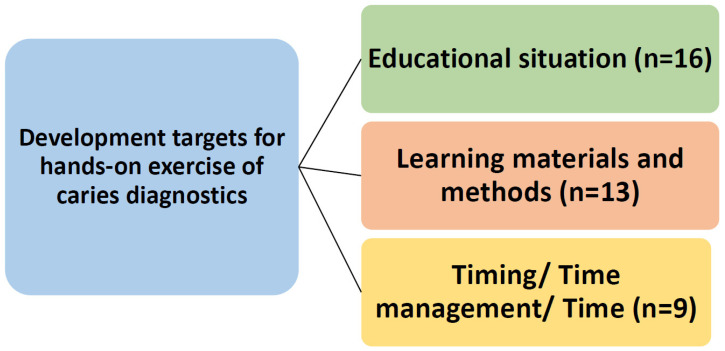
The topics to be developed in the hands-on exercise for third-year dental students in learning caries diagnostics.

Figure 1, Figure 2 and Figure 3 present the subcategories based on the feedback. The exercise offered a communal learning experience and additionally diverse working methods, materials, and detection methods. Learning caries detection was promoted by topics shown in Figure 1 using various radiographic and clinical photographs, versatile and independent working methods, and demonstration of caries lesions by splitting the extracted teeth. Interaction between the students and teachers was reported to be beneficial, too. Authenticity and the number of teeth allowing repetition were also perceived as promoting learning (Figure 1).

The most challenging parts of the exercise were the diagnostics itself, the methods, and the learning situation. The situation was considered taxing with many repetitions. Due to the long duration of the exercise, it was difficult for some students to maintain concentration. It was also difficult to identify and distinguish between ICDAS classes, i.e., to assess both activity and depth. Interpretation and evaluation of clinical and radiographic photographs was not considered easy, nor was the use of FOTI (Figure 2).

The fewest answers were given to the question regarding suggestions for improving and developing the exercise (*n* = 38). However, the students provided some developmental suggestions, i.e., teaching different diagnostic methods individually by hand (FOTI, instruments). Another suggestion was to include various sample teeth, for example, to learn to use different methods, i.e., FOTI. In particular, the students would have liked more time to complete the exercise (Figure 3).

## 4. Discussion

This qualitative study showed that dental students perceive the hands-on exercise on caries diagnostics as both a communal and individual learning experience. Almost everyone found practical exercise with the authentic, extracted teeth useful and necessary. The diverse methods used in the exercise complemented each other well, demonstrating various degrees of caries lesions. A benefit of such exercise was the number of repetitions. On the other hand, the exercise was considered taxing, and the exercise did not allow everyone to absorb all information during the session.

This study utilized inductive qualitative analysis developed to analyze previously uninvestigated phenomena consisting of several different components [10]. The method offered a new perspective to the teaching situation, allowing a meaningful categorization of the information obtained from feedback. Qualitative analysis thus provided important information not possible to achieve in any other way that can be utilized in the development of the teaching method in the future. To the best of our knowledge, this is the first qualitative analysis on teaching caries diagnostics to dental students. Instead, the method has been used in the past in analyses of medical education to find ways to support young teachers as they develop medical education [11].

Previous studies have quantitatively assessed the learning experience on the topic by, for example, investigating how well introduced criteria for detection of caries lesions in dental teaching have been adopted by the students [7]. This was not studied here. On the other hand, based on the research results of Zandona and colleagues [12], previous clinical experience has little relevance in learning new criteria. In their study, after lectures and hands-on exercise, there was no significant difference in the diagnostic outcomes of dental students and teachers and graduate dentists. However, it has been reported that it is difficult for both dental students and newly graduated dentists to find incipient rather than advanced lesions [7,13]. It is equally important to keep in mind that dental education is the combination of theoretical knowledge, hands-on trainings, and practices. Implication of these strategies significantly improves learning performance [14]. Learning to identify different stages of caries lesions was one of the main aims in this exercise, which, however, was not included in the analyses.

Detection of incipient lesions is important, as caries treatment increasingly focuses on controlling caries [5]. The classification of lesions has only become more common in the 2010s, which is why previously graduated dentists have no or only little knowledge of the classifications and have to learn it through postgraduate training. However, experienced dentists have the knowledge that the work brings to assess a patient’s level of caries risk and estimate the lesions. Zandona and colleagues [12] report that dentists with more experience do not end up with restorative treatment in their treatment plan as often as their newly graduated counterparts. Classification of lesions according to ICDAS criteria facilitates decision-making for students and recent graduates, but most likely also benefits experienced dentists.

Almost all the students found the depth and activity exercise of caries lesions, both with photographs and authentic teeth and different additional measures, good as an adjunct to the lectures. The exercise was perceived as useful, instructive, and necessary. Thus, the aim to combine theory and practice to promote learning was achieved. The research findings are in line with the conclusions of El-Damanhoury and colleagues [13], when they reported that comprehensive caries diagnostics education requires both theoretical and practical learning supporting each other. The place of this teaching session in the curriculum or dividing it into several courses should be considered. A refresher course at the end of dental studies might be helpful. This kind of teaching could also be used to introduce caries risk assessment, treatment planning, and deciding about recall intervals [14].

Interestingly, the exercise combined individual and communal learning. The materials and methods were appreciated. Most likely, the exercise will enable learning for different kinds of learners. Having a chance to work independently and to do things with “their own hands” was also emphasized in the students’ feedback, which means that doing practical implementation of theoretical knowledge promotes the learning process. The results encourage the continuance of this kind of exercise with authentic teeth in the future. At present, Finnish law allows this, as mentioned in the ethical section [15].

Regarding the learning situation, the reports emphasized the difficulty to remain focused throughout the exercise. It is possible to address this by giving enough time and opportunity for reflection on learning. In addition to people’s learning differences, prior background knowledge also influences the acquisition of new knowledge. McGleenon and Morison [16] also highlighted that students are the most confident with skills in which they have prior experience. Despite a brief lecture before the exercise summarizing the main topics, it was not enough for all, and some did find the exercise difficult. The most challenging aspect of caries diagnostics was related to identifying and distinguishing between the ICDAS categories. In the future, it is worth thinking about ways to support those with poor basic knowledge in the exercise. For example, it could be useful for students to review caries diagnostic lectures independently, followed by peer-based collaborative learning and finally reviewing the exercise. Hands-on exercises on dental caries diagnosis for dental students are important for understanding and differentiating the most suitable treatment options (invasive or noninvasive) and progression (active or arrested).

The fewest answers were given to the question regarding suggestions for improving the teaching situation (*n* = 38). The students hoped that various diagnostic methods should be taught in even more detail, e.g., FOTI and probe. Regarding photographs, more attention should be paid to their good quality. It is important that all photographs are available even after the exercise for rehearsing. Students also wanted more time to complete the exercise, as, for example, some students did not have time to go through all the diagnostic methods properly. More time is needed, especially if new caries diagnostic methods are introduced. Teaching in smaller groups or collaborative learning could be useful, as well.

The strength of the study is that, although the study population was small, the sample size was sufficient for qualitative analysis, because the size of qualitative research data is sufficient when saturation is achieved [10]. Furthermore, students examined premolar and molar teeth during the hands-on exercise. In this study, saturation was achieved for all questions, i.e., the content of the answers began to repeat the same things that had already come up in previous answers. In addition, the material was of good quality. It described the experiences of the students in various ways. The topic of the study is important because, to our knowledge, no similar qualitative studies have been conducted before. The timing for the exercise was good, just before the start of clinical patient work, when students are motivated to learn practical things like caries diagnostics.

A practical exercise is one option for improving the learning experience of dental students in caries diagnostics in addition to lecturing. In the future, we must strive to consider the diversity of students as learners in dental education as well as aim for deeper understanding. An exercise introducing new criteria can also increase the knowledge of previously graduated dentists in the future by using an online exercise based on photographs, which were found useful. In this way, a practical exercise would provide didactically important information for pre- and postgraduate dental teaching.

## 5. Conclusions

In conclusion, this qualitative study showed that, despite the challenge in caries diagnostics, dental students perceive the hands-on exercise as both a communal and individual learning experience.

## Data Availability

Data are available upon request.
